# Wild Orangutan Males Plan and Communicate Their Travel Direction One Day in Advance

**DOI:** 10.1371/journal.pone.0074896

**Published:** 2013-09-11

**Authors:** Carel P. van Schaik, Laura Damerius, Karin Isler

**Affiliations:** Anthropological Institute and Museum, University of Zurich, Zurich, Switzerland; Midwestern University & Arizona State University, United States of America

## Abstract

The ability to plan for the future beyond immediate needs would be adaptive to many animal species, but is widely thought to be uniquely human. Although studies in captivity have shown that great apes are capable of planning for future needs, it is unknown whether and how they use this ability in the wild. Flanged male Sumatran orangutans (*Pongo abelii*) emit long calls, which females use to maintain earshot associations with them. We tested whether long calls serve to communicate a male's ever-changing predominant travel direction to facilitate maintaining these associations. We found that the direction in which a flanged male emits his long calls predicts his subsequent travel direction for many hours, and that a new call indicates a change in his main travel direction. Long calls given at or near the night nest indicate travel direction better than random until late afternoon on the next day. These results show that male orangutans make their travel plans well in advance and announce them to conspecifics. We suggest that such a planning ability is likely to be adaptive for great apes, as well as in other taxa.

## Introduction

It seems obvious that animals, just like humans, would benefit from being able to plan, i.e. to follow a self-determined goal over a longer time period (i.e. beyond its current motivational state [1], [2]). This way, short-term motivational states such as hunger would perhaps briefly divert but not dissuade the animals from pursuing this superordinate goal. Optimal exploitation of a landscape's resources often requires movements between different areas in specific time periods. Similarly, finding a mate may require moving to distant locations where the presence of the opposite sex can be anticipated. Planning for the future beyond immediate ecological or social needs should therefore generally be favored by natural selection. However, despite these potential benefits, the evolution of this ability may be prevented due to the caloric costs of the increased brain size needed to implement the requisite cognitive capacity [3]. Moreover, most animals probably manage well without the planning ability. First, reliance on a set of innate rules to respond to a variety of environmental cues may often be sufficient to achieve near-optimal scheduling of maintenance and social activities and space use, even on time scales well outside current motivational states, such as long-distance migration [4]. Second, cognitively simpler mechanisms, such as associative learning, may be equally effective [5].

Planning for the future is therefore expected only in a subset of animals. First, the ability should be selectively favored where the target location changes frequently and unpredictably and travel costs are high. A high efficiency in range use is especially at a premium because animals have only a limited scope for increasing their foraging effort without reducing their net daily return [6]. Second, animals must be able to bear the energetic costs of the brainpower needed for such a high-level cognitive ability. Thus, species that are already relatively large-brained may have a head start in evolving the ability to plan ahead. Indeed, the ability to plan for future needs has long been considered uniquely human [7]. This ability critically relies on two cognitive abilities: self-control and mental time travel [8]. Self-control is the ability to suppress immediate responses and thus delay reaping a reward, if necessary for long periods of time. Mental time travel, the capacity to construct mental experiences of potential events, critically involves the presence of episodic memory, the recollection of specific events [9]. This episodic system is active when the brain is at so-called wakeful rest in humans [10] and probably chimpanzees [11].

The question is whether this ability to plan for the future is indeed limited to humans. Recent experiments have provided evidence for the presence of both self-control [8], [12] and episodic memory, the two key ingredients of planning for the future, in a range of animal species [13]–[16]. These results suggest that planning for the future may also be found in nonhuman species, especially wide-ranging and highly encephalized ones. Indeed, several recent experiments and observations have shown that some animals, including great apes, possess the ability to plan for future needs [1], [2], [8], [17], [18]. A zoo chimpanzee was observed to cache stones and pieces of concrete before the zoo opened for later use as ammunition on visitors [18], and to even hide them from view to avoid detection [19]. In experiments, chimpanzees and orangutans chose the correct tool to get a reward one hour later [8]. Bonobos and orangutans selected, transported, and stored appropriate tools to use them up to 14 hours later [1]. Western scrub-jays stored food in a place where they had learned they would have access to it the next morning, and when provided with two different food types would store more of one type in a room where they knew only the other food type would be available the next day [2]. They and Eurasian jays also anticipated their own future needs independent of their current motivational state by caching those food items they know they would need by the time they would be able to recover them, rather than the ones they preferred at the time of caching [17], [20]. These results have cast doubt on the hypothesis that the ability to plan for future needs is uniquely human. This ability may be present at least in some (relatively large-brained) corvids and great apes, but perhaps also in rodents [21].

Although experimental evidence is both easier to obtain and meets more stringent standards in captivity, it is important to examine the occurrence of planning for the future in wild animals. It is possible that the presence of such a peculiar mental ability is a mere byproduct of selection on other domain-specific or domain-general cognitive abilities [22]. Thus, to understand the evolution of cognition, we must examine the use, and if possible adaptive significance, of cognitive skills in natural conditions. Although planning in the context of range use is certainly not the only adaptive higher cognitive skill in animals, it may be much more widespread than others such as tool use or intentional communication. Therefore, evidence for long-term planning in one wild-living species would be important as a proof-of-principle that high-level cognitive abilities can indeed be adaptive, rather than being mere spandrels, and stimulate future research in other species.

To investigate whether there is evidence for long-term planning in wild animals, we best start looking in those species that have already demonstrated planning abilities in experimental conditions. Non-human great apes are large and partly or wholly arboreal, range semi-nomadically in large home ranges, and often live in dispersed social systems. Therefore, they face high travel costs to approach their highly versatile targets, and thus meet the kinds of conditions where planning for future social and subsistence needs may be adaptive. However, despite decades of intense fieldwork on great apes, it remains unknown whether they actually use this ability to plan for future needs in natural conditions. A major obstacle is that it is very difficult to rule out the influence of environmental cues without cumbersome field experiments. For instance, chimpanzees carry scarce tools to nut-cracking sites that are out of view [23]. This looks like planning for future needs, but because they do so when traveling toward the nut trees, the animals may already be motivated to crack nuts, allowing for the possibility that associative learning (tools and nuts go together) established the habit. This problem may largely disappear when animals communicate their ever-changing travel patterns to others hours before they execute them.

The present study capitalized on this situation by examining the extent to which the direction of long calls emitted by male Sumatran orangutans (*Pongo abelii*) indicated the direction of their future travel. These orangutans live in dense tropical forests and are semi-solitary, and thus often out of visual contact from others in their population [24]. Sexually mature males may, after highly variable periods of time [25], grow cheek flanges (wide cartilaginous pads at the sides of their face [26]). The average flanged male at our study site (Suaq Balimbing) travels about 1000 m per day in a home range of at least 2,000 ha [27]. Flanged males emit loud vocalizations, audible for over 1 km distance, known as long calls, on average about four times per day [28]. Previous work has shown that long calls reveal the caller's identity [28], [29] and that spontaneously produced long calls differ acoustically from long calls elicited by the presence or activities of other flanged males [29]. Long calls repel lower-ranking males, while attracting higher-ranking males [30], [31]. However, they also attract sexually active females and are often used by females with dependent offspring to remain within earshot of the dominant male, probably because this reduces harassment by other males [31], [32].

**Figure 1:**
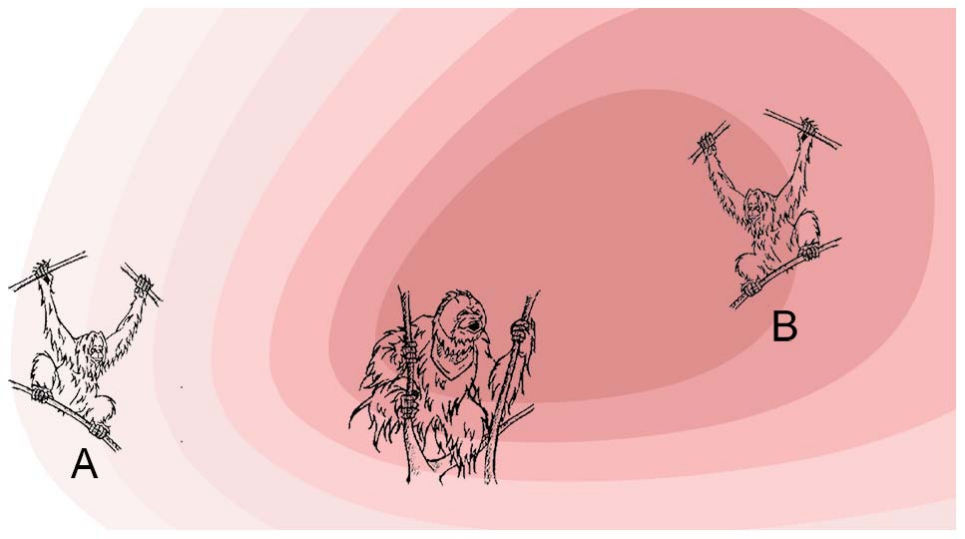
Orangutan males give long calls to attract females and repel rival males. To reach the calling male or remain within earshot of him, female orangutans should move in the direction of the flanged male if they hear his long call only faintly. Due to the large cheek flanges that probably act as a megaphone, to female A the call is not as loud as to female B, even if they are at the same distance from the calling male, and A should move in the direction of the male whereas B need not. However, this system works best if the male actually travels in the same direction as he is calling.

Calling males face in one particular direction during the entire long call. Because only fully flanged males emit long calls, the function of the flanges may be to concentrate the sound energy in the direction of the call. As a result, the perceived distance of the calling male to a listening female will be affected by the angular difference between the direction of the call and the line connecting caller and listener (Fig. 1). Thus, a long call perceived as faint by a listening female (say, female A in Fig. 1) could mean that the male is far away, or alternatively, less far but calling in a direction away from the female. In both cases, a female must approach the call to reach him or remain within earshot with the least travel effort, but only if he consistently travels in the direction he calls. Given the practice of earshot association [31] and because sexually active females sometimes go to calling males [32], we therefore expected that flanged males, if they have a target location or area in mind, give spontaneous long calls in the direction they are later travelling.

Such consistent travel in one direction is unlikely to be an artifact of the direction of movement during calling. Sumatran orangutan males are strictly arboreal, and usually give their long calls when stationary, e.g. during feeding, sitting, or resting on a nest. They face consistently in one direction during the entire call, which lasts about 80 seconds on average, but may last up to 4 minutes [28]. It could be argued that the male initially travels simply in the direction he was facing. However, during a single feeding bout in a tree, the male faces in many different directions. Moreover, when he calls during rest, it would be extremely unlikely that he can maintain travel in the direction he faced during the call for more than a few minutes because he cannot travel in a straight line from tree to tree. Instead, the male must look for branches that can hold his weight, creating a meandering route, albeit one with an overall direction. Hence, if males actually continue to travel in the direction of the long call for a long period of time despite interruptions, such as overnight rest, and do so despite the fact that their target location or direction changes from day to day, this would indicate that they plan their travel routes in advance [1].

Here, we first test whether the direction in which flanged male Sumatran orangutans give spontaneous long calls generally predicts the subsequent travel direction. Second, we investigate whether a new spontaneous long call indicates the subsequent travel direction better than the old one would have, if no new call had been given. Third, we test the extent to which long calls given in the evening at or near the night nest still indicate travel direction during the next day, thus indicating future planning independent of the current motivational state.

In addition, we examine whether the audience actually uses the information contained in the calling direction by adjusting their travel routes. Because animals are not active during the night, and thus cannot be distracted by other cues or social activity, we selected the response to long calls heard the evening before. If during the following morning, they adjusted their travel direction, and did so, before a new long call could affect it, this would strengthen the adaptive interpretation of the announcement of travel plans by the flanged males.

## Methods

### Data collection

The data were collected in Suaq Balimbing (03°04′N, 97°26′E), a peat swamp forest located within the Kluet portion of Gunung Leuser National Park, Nanggroe Aceh Darussalam province, Sumatra, Indonesia. The Indonesian Institute of Sciences, LIPI, granted permission to do research in Indonesia, and the directorate general of Nature Conservation and Forest Protection, PHKA, gave permission to work in Gunung Leuser National Park.

Local orangutan density in the study site is about 7 ind/km^2^ [24]. The data on flanged males analyzed here were collected from April 1994 through August 1999. Every encountered individual was followed from nest to nest (∼10 to 12.5 hours/day) for a maximum of ten consecutive days. Observers always kept a distance of at least 5 m and never attempted to interact with the subjects. The travel routes of the focal animal and its location at each half hour point (i.e. at full and half hours) were indicated on 1∶7,000 maps of the study area. Locations were estimated relative to a dense trail system, in which each trail was marked every 50 m, using pacing and compass directions. Comparison of independently produced travel maps indicated inter-observer differences in location of <25 m [33]. The average distance covered by a flanged male during a full day was almost exactly 1 km.

General behavioral data were collected according to established standards and methods [34] by multiple well-trained observers, whose inter-observer reliability was high [34], [35]. For each long call emitted by the focal male, we recorded the time and the direction he faced when calling using a compass. If the long call was immediately preceded by a physical or social disturbance, such as calls from other adult males, the activities of conspecifics within 50 m, or loud noises in the forest (usually the result of tree falls), it was classified as *elicited*. Many tree falls are caused by flanged males, who respond to a social disturbance by pushing over large dead snags, dead standing trees [24], [36]. All other long calls were classified as *spontaneous*.

### Data analysis

A sample of 1169 long calls given by 15 different flanged males on 320 follow days was analyzed; the dominant male contributed 696 of these (on 152 days).

**Figure 2:**
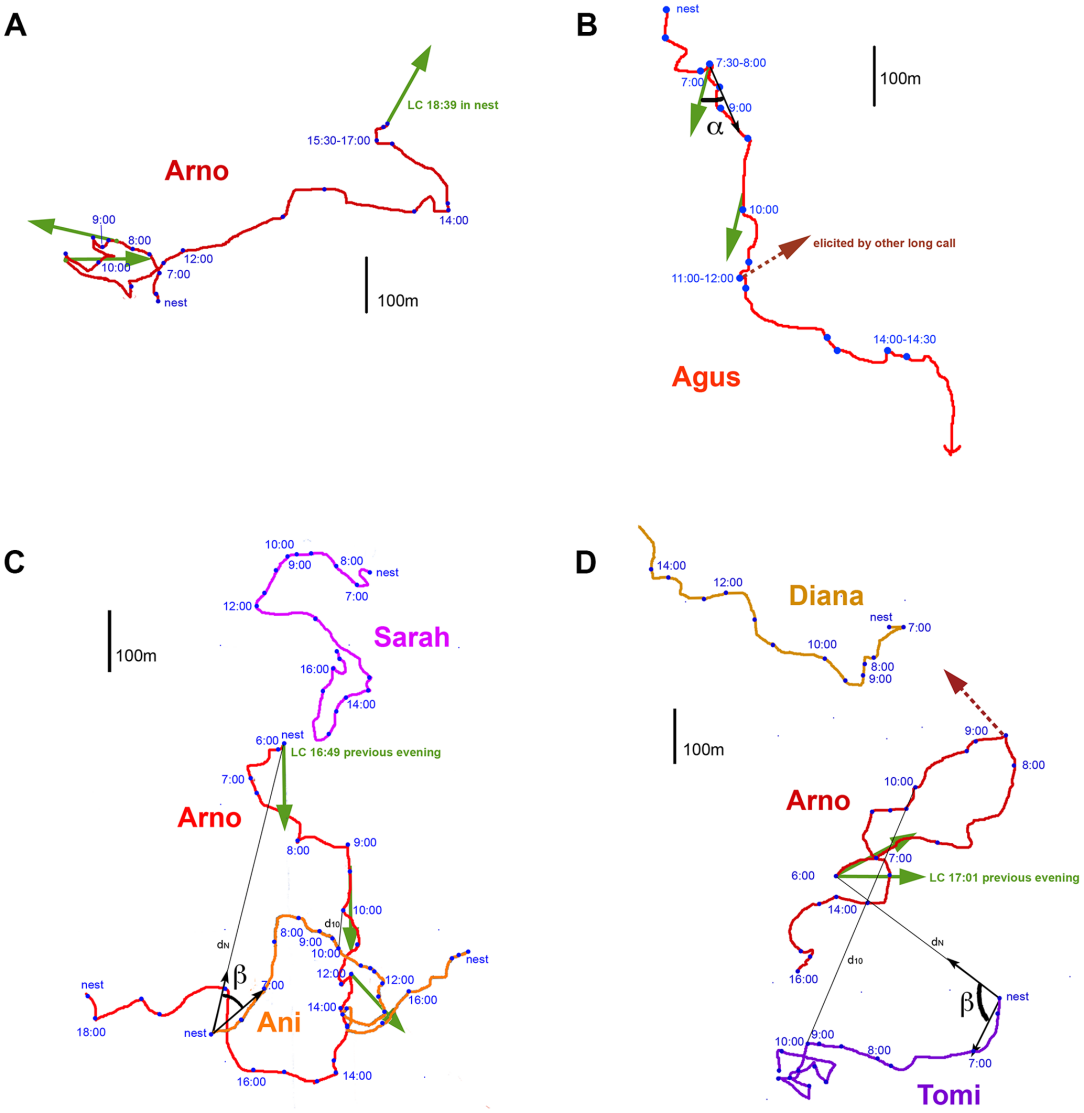
Travel maps showing travel routes and long calls of wild orangutans. Spontaneous long calls are indicated by green arrows, elicited long calls by red broken arrows. The deviation angle a between the long call direction and the travel direction was measured in 0.5-hour steps. Travel routes of flanged males: A) Arno (dominant male), 17 January, 1995; B) Agus, 8 April, 1995. Travel route of the focal animal and conspecifics the day after a long call was given shortly before nesting and from the nest area: C) Arno and the females Sarah and Ani, 8 August, 1994; D) Arno, the female Diana and the male Tomi, 15 April, 1996. For every individual, we measured the angle β between the direction in which the evening long call of the flanged male was heard, and the direction in which it took off from the nest the following morning. The distances between the two individuals were measured at the night nest and at 10 AM (d_N_, d_10_).

**Figure 3:**
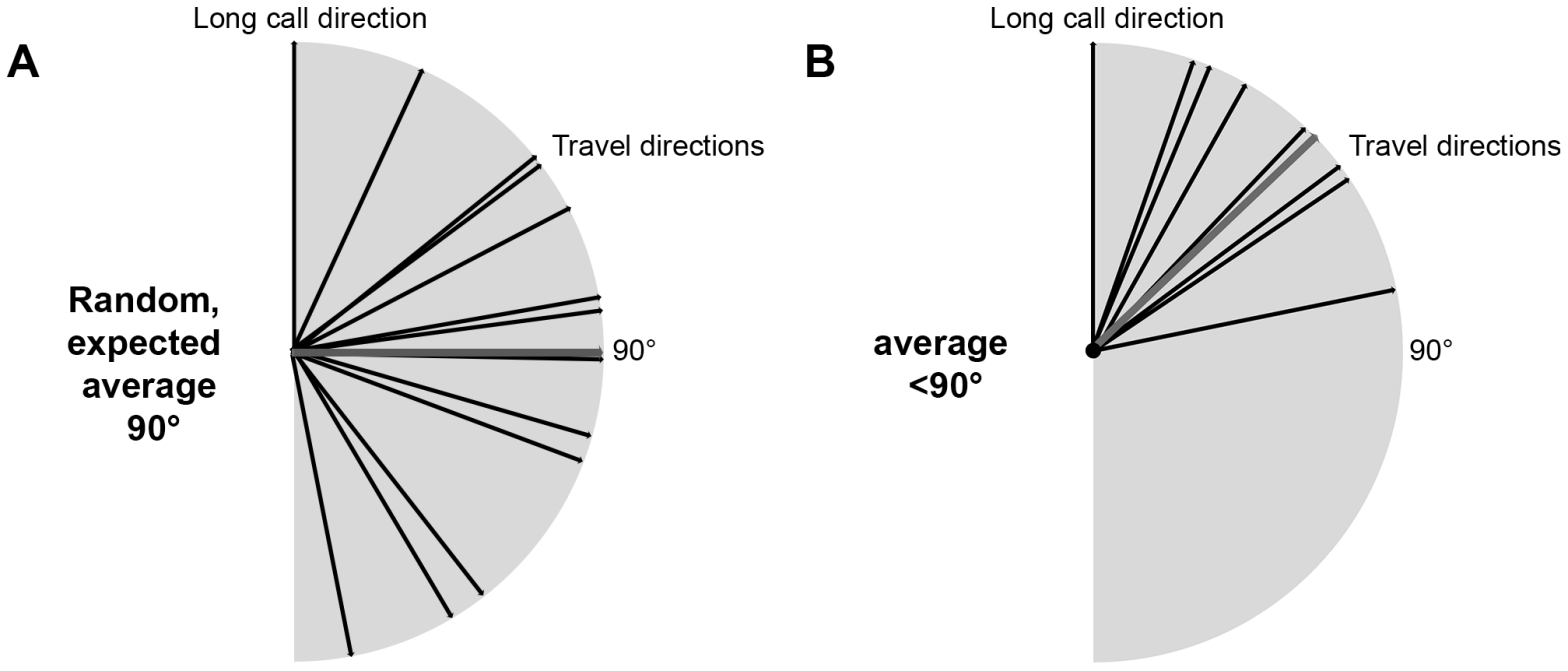
Relation between long call direction and subsequent travel directions. A) If travel direction and long call direction are independent of each other, we expect a uniform distribution of the deviation angles. The expected average deviation angle (in grey) would be 90°. B) On the other hand, if long call directions predict subsequent travel directions, the expected average deviation angle is significantly smaller than 90°.

Travel direction was measured from the travel maps relative to the male's location at the time of the long call, for each of the male's subsequent locations, in half-hour intervals, until reaching the night nest (see Fig. 2a and 2b for examples). For each subsequent location, we measured the deviation angle, α, between long-call direction and travel direction. Because deviations to the left or the right were not discriminated, angles were between 0° and 180°. Both the long call directions and the travel directions in our sample are approximately uniformly distributed between 0 and 360° with a median of 180°. The uniform distribution of travel directions is reasonable because there are no obstacles to movement, such as rivers, that would lead to directional biases within this homogeneous habitat. If travel direction is independent of long call direction, the average deviation angle in a large sample is expected to approach 90° (Figure 3), which is thus our null model. Because consecutive long calls by the same individual need not be independent of each other, we used only the first long call per day of any individual (N = 263), to test whether long-call direction predicts travel direction, provided at least two hours of observation after the long call were available.

As the calling direction of long calls given during moving could be influenced by the male's current travel direction, we repeated all analyses including only those long calls given while absolutely stationary (N = 245 long calls). This did not affect the levels of significance of the statistics, and results are therefore not reported.

To consider the effect of variation between individuals, we used general linear mixed models (GLMM) in JMP version 9 [37], with the deviation angles of a given time step as dependent variable, and individual nested within male morph (dominant male vs. other adult males) as a random effect. Due to the imbalance in sample size among the 14 non-dominant male individuals, the proportion of variance explained by this random factor was often negative, indicating a problematic model structure. In addition, especially in analyses with a small overall sample size the commonly advocated REML procedure [38] did often not converge on a solution, forcing us to apply the Method of Moments. As such problems prevent any reliable conclusions from the respective mixed model analyses, and obtaining a more balanced dataset is impossible for studies of wild orangutans, we additionally employed two alternative statistical methods that disregard the differentiation between within-subject and between-subject variation. First, the random factor was simply omitted from the model, yielding a linear model (LM). Second, we applied the more robust two-tailed Wilcoxon signed-rank test. In all cases where the GLMM yielded a positive value for the proportion of variation explained by the random factor, the resulting p-values did not differ in the level of significance from this second approach, the Wilcoxon test (cf. Tables 1, 2 and 3). Therefore, we are confident that the statistical results of the latter can also be considered reliable in the other, more problematic cases.

**Table 1:** Deviation angles between long call direction and travel direction after the first spontaneous long call of a day.

			p-values			Median		
Time after LC	N	%var	p GLMM	p LM	p Wilcoxon	All males	Dominant male	Other adult males
1 h	183	0.048	<0.0001	<0.0001	<0.0001	45***	49***	34***
2 h	206	9.3	<0.0001	<0.0001	<0.0001	55***	59***	42**
3 h	198	3	<0.0001	<0.0001	<0.0001	59.5***	57***	60**
4 h	181	12.8	<0.0001	<0.0001	<0.0001	63***	63.5***	61*
5 h	146	0.8	<0.0001	<0.0001	<0.0001	59***	64***	58**
6 h	115	15.7	<0.0001	<0.0001	<0.0001	62***	65.5***	52**
7 h	84	10	0.009	0.002	0.002	70.5**	75*	60
8 h	60	13.6	0.022	0.024	0.031	78.5*	84	60
9 h	41	<0		0.15	0.259	95	95	89.5
10 h	21	<0		0.815	0.75	107	97	

**Note.** The table lists p-values of three different tests to evaluate whether deviation angles a between long call direction and subsequent travel direction differ significantly from 90°: a mixed-model (GLMM) with the random factor individual[male morph], a linear model (LM), and a non-parametric Wilcoxon signed-rank test (Wilcoxon). For the GLMM, %var denotes the percentage of variance explained by the random factor individual nested within male morph. If this value is smaller than zero, the GLMM result would not be reliable and is thus omitted (see Methods). The median deviation angles are listed for dominant male and the other adult males separately. For the median values, stars denote the level of significance (Wilcoxon): *: p<0.001, **: p<0.01, ***: p<0.001.

**Table 2:** Deviation angles between long call direction and travel direction after the first elicited long call of a day.

			p-values			Median		
Time after LC	N	%var	p GLMM	p LM	p Wilcoxon	All males	Dominant male	Other adult males
1 h	53	21.6	0.001	<0.0001	<0.0001	55***	37.5**	69*
2 h	57	8.3	0.001	<0.0001	<0.0001	61***	66.5**	49**
3 h	49	23.5	0.009	0.002	0.002	66**	73*	55.5*
4 h	44	24.6	0.125	0.063	0.066	70.5	79	55
5 h	37	22.9	0.205	0.132	0.141	78	85.5	46
6 h	31	31.2	0.37	0.449	0.466	84	88	45.5
7 h	19	<0		0.597	0.561	81	99	53
8 h	13	<0		0.4	0.386	70	82	
9 h	11	<0		0.916	0.966	82		

**Note.** For details, see Note of Table 1.

**Table 3:** Deviation angles between direction of the last spontaneous long call given shortly before nesting and travel direction on the subsequent day.

			p-values			Median			
Clock time next day	N	%var	p GLMM	p LM	p Wilcoxon	All days	Subset 1	Subset 2	Subset 3
7:00	39	<0		0.071	0.042	70*	48*	46	85
8:00	41	<0		0.002	0.001	49.5**	46.5***	40**	60
9:00	39	<0		0.005	0.003	51.5**	47***	44*	65
10:00	39	<0		0.034	0.015	62.5*	52**	49.5*	62.5
11:00	37	2.2	0.078	0.005	0.004	53**	42***	44*	52
12:00	36	17.4	0.014	0.002	0.002	53**	41***	43.5*	49*
13:00	35	28.9	0.032	0.002	0.001	50**	43***	43.5**	37.5*
14:00	33	<0		0.008	0.007	59**	54**	46*	54*
15:00	30	11.3	0.200	0.032	0.025	62.5*	43*	39*	50.5*
16:00	24	16.6	0.082	0.033	0.024	50*	54*	46*	50

**Note.** For details, see Note of Table 1. The median deviation angles are listed for all days, and subsets 1–3 separately. Subset 1 includes only those 26 days on which no new long call was given the next morning before 8:00 AM (Subset 2: before 9:00 AM, N = 18 days). Subset 3 includes only those 18 days in which the evening long call was not given in the direction of travel during the last hour before nesting on that day.

With the above procedures, we tested whether, for any given time step *t* after the long call, the deviation angles α*_t_* differed significantly from 90°. As the travel map recorded locations at every 30-minute point regardless of long call activities, the first angle α_1_ was measured at the first 30-minute point, i.e. between 1 and 29 min after the long call, the second angle α_2_ was measured at the second 30-minute point after the long call, etc. Then, we tested whether at a given time step, say 5 hours after the long call, the deviation angles of all analyzed long calls differ significantly from 90°. This test was repeated for each time step (with decreasing sample size) to estimate the maximum time interval during which the long calls still indicated travel direction better than random. We did not apply corrections for repeated testing because this test series does not represent cumulative evidence for our hypothesis. Note that the sample sizes for the different time steps are not equal, as the animals were observed for varying time spans after the first long call of the day, until nesting in the evening or until they disappeared from sight. Also, if the long call was given during rest, the first deviation angle could only be measured after the animal had changed its location. Therefore, the maximum sample size was usually obtained for the second hour after the long call was given (cf. Tables 1 and 2).

In pairs of consecutive long calls on a given day, the second long call may be a better indicator of travel direction than the first one would have been if the second long call had not been given. To examine this possibility, we analyzed pairs of consecutive long calls (N = 374 pairs, 222 of them by the dominant male). For this data set, we tested the following hypothesis: If a male gives a new spontaneous long call when he has deviated from his planned travel direction to announce his new plan, the deviation angles of the old call should be larger than the deviation angles of the new call during the period following the new long call (to compare it with the information available to the audience had the new call not been given). In this test, we used the average of four consecutive deviation angles after the second long call to correct for brief turns and estimate the overall direction. We again used GLMM with individual within male morph as random effect, but the proportion of variance explained by the latter was negative, and we therefore report the results of two-tailed Wilcoxon signed rank tests.

To test whether evening calls given immediately before nesting still predicted travel direction after overnight rest on the following day, we analyzed long calls that were given less than 30 min before nesting and followed by not more than one moving bout before settling down in the night nest. In total 48 long calls were available for this analysis, 41 of which were spontaneous (33 and 30, respectively, were given by the dominant male).

Finally, to test how conspecifics reacted the following day to long calls given before nesting the previous evening, we compiled travel trajectories of all individuals that were followed in the study area on the relevant days, and selected those that were less than 1 km away from the male when he gave the long call (see Fig. 2c and 2d for examples). First, for each pair of individuals, the angle β between the direction of movement during the first hour after leaving the nest in the morning and the direction from which the other individual heard the long call the previous night was noted. We predicted that females would tend to travel towards the direction in which they heard the long call and thus the deviation angle β would be less than 90°. Second, the distance between the male who gave the long call and its conspecific was measured both at the night nest (d_N_) and at 10 AM the following morning (d_10_). We predicted that subordinate males would increase their distance, and thus d = d_10_−d_N_ would be greater than 0.

## Results

### Does call direction of spontaneous long calls predict subsequent travel direction?

**Figure 4:**
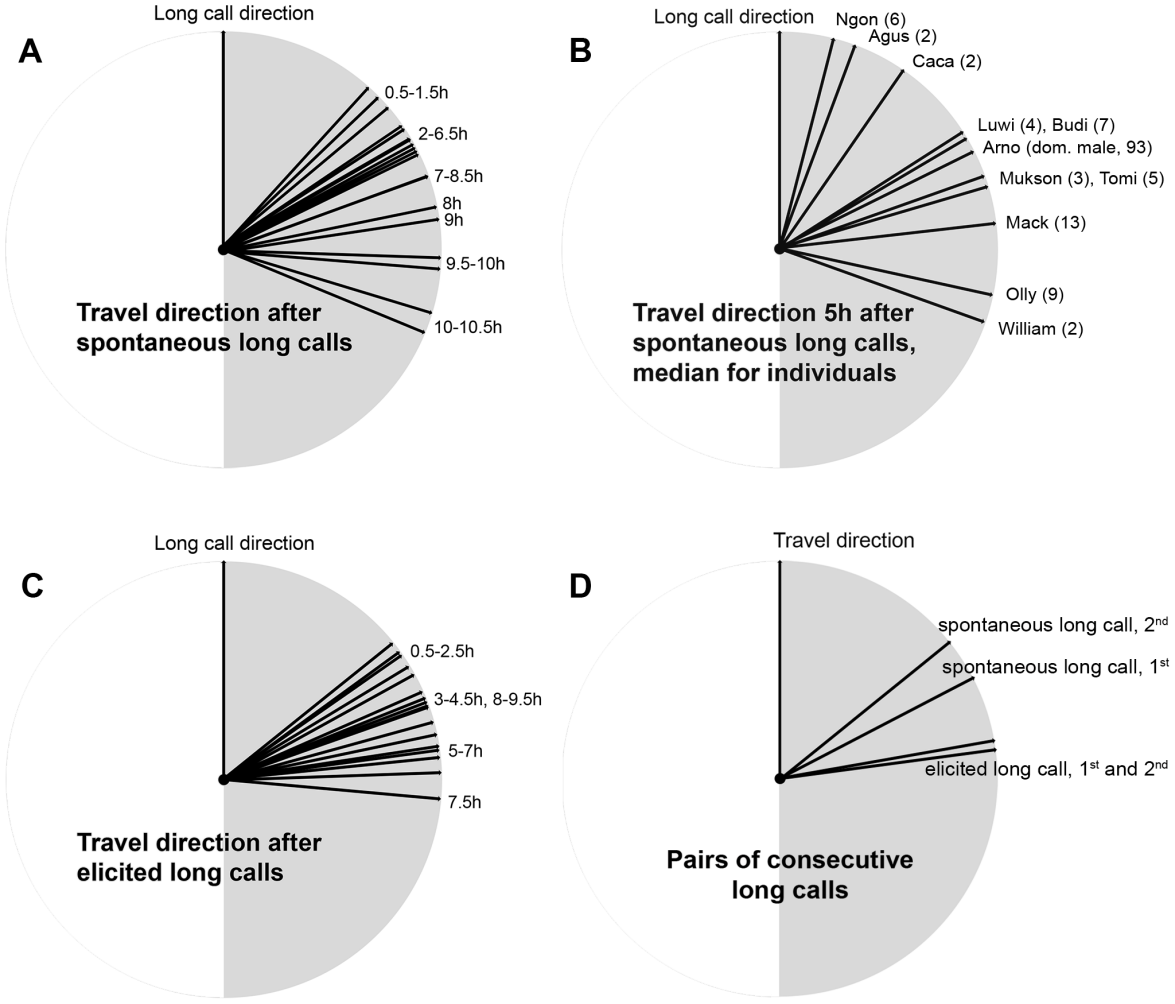
Observed deviation between long call direction and subsequent travel direction. Median deviation angles for each time step are shown on a circular scale. A) On the same day, after the first spontaneous long call of a day. Time is number of hours elapsed after the long call is given. Statistics are given in Table 1. B) Variation between individuals: median deviation angles for each male, 5 h after a spontaneous long call. Sample size for each male is given in parentheses. C) On the same day, after the first elicited long call of a day. Time is number of hours elapsed after the long call is given. Statistics are given in Table 2. D) Differences between pairs of consecutive long calls: the original and a subsequent call. The subsequent (second) spontaneous long call predicts travel direction better than the original (first) spontaneous long call. If the second long call is elicited, however, it does not improve the prediction of travel direction. The deviation angles are averaged over four half-hour time steps after the second long call. Statistics are given in the text.

Figure 4A shows the deviation angles between calling and travel direction (α) for every half-hour time step elapsed since a spontaneous long call was given. If the male orangutan's directions of calling and travel were independent, the expected deviation between long call and travel direction would have an average value of 90°. Following the first spontaneous long call of the day (N = 206), deviation angles were significantly lower than 90° for 8 hours (Tab. 1).

Figure 4B illustrates the variation between individuals by showing the median deviation angle for each male 5 h after a spontaneous long call. The pattern for the dominant flanged male in the area, with by far the largest sample size, is not significantly different to that of the other males (GLMM, N = 146 long calls, p-value of male morph = 0.755, 0.8% of variance explained by individual within male morph).

Orangutan females can distinguish elicited long calls from spontaneous ones [29], and elicited calls are generally given while the male faces in the direction of the disturbance, and thus less likely to indicate future travel direction over long periods of time. Indeed, the deviation angles of elicited long calls (N = 57) were only predictive of the travel direction for up to 3.5 hours (Tab. 2, Figure 4C).

### Does a new spontaneous long call signal a change in travel direction?

A flanged male often gives more than one spontaneous long call on a given day. If spontaneous long calls represent the main travel direction or his destination, a new long call may indicate that the male has changed his long-term travel direction. We therefore examined pairs of successive long calls given by the same male. We compared the deviation angles following the second long call to those of the first long call during the same period of time (both averaged over four half-hour points after the second long call). If orangutans announce a change of main travel direction, the new long call should indicate travel direction better than the old one would have done, if no new long call had been given.

The results show that the second long call's average deviation angle is significantly smaller than that of the first long call during the same time period if the second long call was given spontaneously (Wilcoxon signed-rank test, mean difference 12.6±3.9°, p_Wilcoxon_ = 0.002, N = 268) but not if it was elicited (mean difference −11.1±6.6°, p_Wilcoxon_ = 0.093, N = 106). If the first long call was elicited and the second one spontaneous, the latter predicted travel direction better by an average of 23.1±8.3° (p_Wilcoxon_ = 0.007, N = 52). Thus, when males emitted a new spontaneous long call, it predicted the subsequent travel direction better than the previous long call would have done (Figure 4D).

### Do spontaneous long calls also predict travel direction after overnight rest?

**Figure 5:**
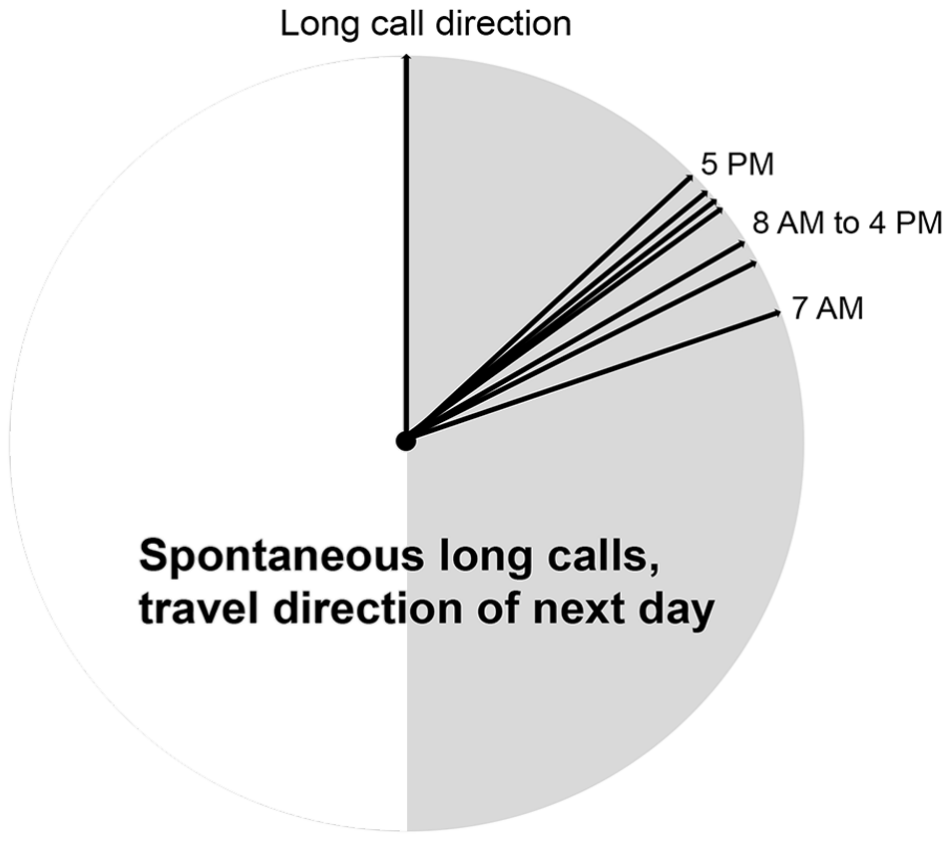
Deviation between evening long call direction and travel direction of the next day. The graph depicts the median of deviation angles between travel direction and the direction of the last spontaneous long call of the previous day, given shortly before nesting or from the nest. Time is clock time during the day following the long call. Statistics are given in Table 3.

The results so far show that long-calling males indicate their main travel direction. However, long-term planning for future needs requires that it is independent of current activities, needs and motivations, i.e. the planning and the target event are separated by a long period of other activities. Because overnight sleep is likely to reflect a discontinuity of mental activity, we also analyzed whether spontaneous long calls given in the evening at or near the location of the night nest (N = 41) indicate the next day's travel direction. Indeed, deviation angles were significantly smaller than 90° until 16:00 hrs the next day (Tab. 3, Figure 5), and remained low until nesting time in the evening. Thus, including overnight periods, long call direction predicted travel direction better than random for around 22 hours, almost a full day. The male does not require any reminders or reinforcement of his original main travel direction, because this result remains the same if he did not give any long calls early the next morning, during the first few hours after becoming active at about 6:00 AM (no new long call until 8:00 AM (N = 26) or until 9:00 AM (N = 18), P<0.05 until 16:00 hrs next day, Tab. 3).

The spontaneous pre-nesting long call indicated the next day's travel direction even if this call represented a clear break with the main travel direction of that day: The 18 spontaneous long calls that differed by more than 45° from the direction of movement during the last hour before nesting nonetheless predicted travel direction reasonably well until 15:00 hrs next day (Tab. 3), despite detours during the morning.

### Does the audience use this information?

It has been demonstrated that, in a short time frame of 30 min to 1 h, adult females generally stay within earshot of long calls given during the day, whereas subordinate males tend to move away [31]. Here, we asked whether conspecifics also used the information from long calls given at or prior to nesting the evening before, by recording their ranging responses during the following day. Including only cases where there were no long calls by the male on that morning before 8 AM that could influence their decisions, we find that the difference between the immediate departure direction in the morning (measured after 1 hour) and the direction from which the focal animal heard the long call the previous night was significantly smaller than 90° for females (Wilcoxon signed-rank test, N = 13, p = 0.046, median 30°) but not for males (N = 7, p = 0.938, median 80°). To assess whether the delayed response mirrored this immediate response, we also measured the distance between the focal male and his audience from the night nest to 10 AM that morning. Whereas females that had heard the call remained at constant distance from the calling male (Wilcoxon signed-rank test, N = 13, p = 0.879, median 20 m), the males that heard it increased their distance (N = 7, p = 0.031, median 150 m). Thus, the audience used the male's long call made the previous evening to adjust their own travel direction the next day. This result is also consistent with the use of some form of episodic memory.

## Discussion

Captive experiments have previously suggested the presence of the ability to plan for the future in great apes, but cannot reveal in which natural context this ability is used, if at all. The present study strongly suggests that wild Sumatran orangutans at Suaq Balimbing use this ability in the range-use context. Thus, the last long call given shortly before the flanged male went to sleep for the night provided a better than random prediction of his travel direction during the next day until 16:00 hrs, hence approximately 22 hours after the call was given. These findings therefore indicate that flanged male Sumatran orangutans make their travel plans at least a day in advance and announce them through their spontaneous long calls. The ranging responses of his audience show that other orangutans actually use this information, suggesting in addition some communication of plans from the male to his female audience. The delay in responses by the audience on the next day to long calls heard the evening before is also consistent with the presence of episodic memory in the listeners.

The flanged males may benefit from doing so because it allows the females in their community to reach them when sexually receptive, but also to remain within earshot with them when not. Even when not sexually receptive, females are often harassed or mated forcefully by unflanged males, who are subordinate to most flanged males [39]. The earshot associations thus enable females to move toward the calling male when harassed by other males [39], [40], and are consistent with the existence of a female mating preference for the locally dominant male [39].

Our study in a wild setting necessarily includes a variety of unrecognized sources of noise in the data, but the underlying pattern remains clear despite the noise and also after several attempts to pre-filter the data (e.g. by excluding long calls given during moving). Potential shortcomings of the human observers such as inaccuracy in determining the calling direction, or wrongly assigning a long call to being spontaneous when it was in fact elicited by something the observer did not hear, could of course obscure the pattern we found, but they could not falsely generate it. Moreover, there is additional independent evidence for planning beyond the current motivational state. The males frequently make a detour in the first hour of observation in the morning, probably to satisfy their immediate needs for food, and only later resume their announced long term travel direction. They do not, however, return to the point at which they made the call the previous evening, excluding the explanation of associative memory based on cues at that location. Finally, although our study includes only one population with one dominant male, given the basic similarities in all the relevant variables of other populations of Sumatran orangutans, it is likely that these results apply to the species as a whole.

In an observational field study, it is impossible to definitively identify the mechanisms responsible for the long-term predictive value of the male's long-calling direction for his travel behavior. The position of the sun, a mental map, or yet undiscovered magnetic orientation abilities [41] may all help the male to find its way in the flat-canopied swamp forest, where orienting on distant landmarks is difficult, especially since male spend most of their time well below the top canopy. However, this distant target changes from day to day, as he roams around his range, with its continuously shifting opportunities for feeding and social interactions. Thus, identifying the mechanism used for orientation does not help to decide whether the male plans for future needs. Indeed, the important point is that a male orangutan is maintaining an internally generated directional choice towards a distant target out of current sensory range, over a prolonged time period, despite meandering routes. He remembers the main travel direction in the face of numerous distractions, sometimes lasting for hours, or even overnight. In contrast to migrating birds or homing pigeons, however, the orangutan occasionally adjusts his plan, most likely responding flexibly to the changing position of conspecifics. Our data shows that a new spontaneous long call predicts travel direction even better and may therefore indicate a change in travel direction. In sum, the observed behavior of male orangutans would be very difficult to explain by simple rules of thumb, and is best described as planning based on episodic memory.

In terms of its mechanistic basis, this planning ability presumably relies on episodic memory, given the experimentally demonstrated ability in great apes to plan for future needs, the detailed mental representation of large areas [42], [43], and the presence of strong spatial memory abilities [43], [44], allowing them to move in straight lines toward distant targets. Taken together, the results of this study and the planning experiments with captive apes indicate that neither the episodic system nor planning are tied to a single goal in nonhuman apes, and thus are not narrow competences in the sense of Premack [45]. Obviously, future work is needed to explore in more detail in what conditions this planning ability is used (e.g. exploitation of food, searching for mates, avoidance of known rivals) and to what extent it is similar to human planning for the future [45]–[48]. Especially the latter question will require novel approaches such as brain imaging.

In conclusion, regardless of the mechanisms used, wild orangutan males indicate future travel directions through their long calls over a span of about one day. This broadcast information is used in turn by their audience to adjust their own range use. These findings strongly suggest the presence of the ability to plan for the future, as demonstrated in captive experiments with the same species. Such an ability is probably adaptive in many ecological and social conditions. Among orangutans, the combination of a patchy, scarce diet (trees with ripe fruit), high energy requirements due to large body size and an expensive arboreal locomotion style [49], and a dispersed social system suggests major benefits for high travel efficiency and thus a long-term planning horizon. We therefore do not expect such a planning ability to be limited to orangutans, but rather to exist also in other apes and perhaps other large-brained animal taxa. However, only the fact that orangutan males happen to announce their plans to conspecifics allowed us to recognize its usage in the wild.
